# Granulomatous Lesions in the Head and Neck Region: A Clinicopathological, Histochemical, and Molecular Diagnostic Study

**DOI:** 10.3390/diagnostics15162055

**Published:** 2025-08-16

**Authors:** Amjad S. Ali, Bashar H. Abdullah

**Affiliations:** Department of Oral Diagnostic Sciences, College of Dentistry, University of Baghdad, Baghdad 10071, Iraq; amjad.ali2306m@codental.uobaghdad.edu.iq

**Keywords:** granulomatous lesions, head and neck pathology, tuberculosis (TB) diagnosis, PCR for *Mycobacterium tuberculosis*, tuberculous granuloma

## Abstract

**Background/Objectives**: Granulomatous lesions of the head and neck arise from diverse infectious and non-infectious causes, with tuberculosis (TB) being a predominant etiology. This retrospective study analyzed 42 cases from the archives of university of Baghdad, College of Dentistry (1975–2025). This study aimed to characterize the clinicopathological features of these lesions and to assess the diagnostic performance of histochemical stains and real-time PCR in identifying infectious etiologies—particularly *Mycobacterium tuberculosis*—in formalin-fixed, paraffin-embedded (FFPE) tissue samples. **Methods**: Definitive diagnoses included 25 TB cases confirmed through clinical, microbiological, and therapeutic follow-up at the Baghdad Tuberculosis Institute, and 17 non-TB cases classified by predefined clinicopathological criteria supported by relevant clinical data. Zieh–Neelsen (ZN), Periodic acid–Schiff (PAS), and Grocott methenamine silver (GMS) stains were employed to identify acid-fast bacilli and fungal organisms. Statistical analysis was performed using SPSS version 26, with significance set at *p* ≤ 0.05. **Results**: The mean patient age was 36.28 years (SD = 20.6), with a female predominance (59.5%). Necrotizing granulomas were identified in 69% of cases and were strongly associated with tuberculosis, which was detected in 59.5% of specimens. ZN staining showed a sensitivity of 16.7% for tuberculosis, while PCR sensitivity was highly dependent on sample age. The detection rate was 33.3% in samples archived for less than 10 years but only 10% in older samples, resulting in an overall sensitivity of 24.0% for tuberculous cases. Langhans-type giant cells were significantly more frequent in necrotizing granulomas and strongly associated with tuberculosis infection (*p* = 0.001). Fungal infections, predominantly aspergillosis, were confirmed by PAS and GMS in 11.9% and 9.5% of cases, respectively, and were confined to non-necrotizing granulomas. The mandible was the most commonly affected site, and soft tissue lesions were significantly associated with necrotizing granulomas (*p* = 0.004). **Conclusions**: These findings underscore the complementary role of histopathology, histochemical stains, and molecular diagnostics in improving the evaluation and diagnosis of granulomatous inflammation in head and neck lesions.

## 1. Introduction

Granulomatous lesions are defined as organized collections of mature mononuclear phagocytes, often accompanied by epithelioid transformation, multinucleated giant cells, and immune cells such as lymphocytes and plasma cells [1]. Granulomas were first recognized approximately 150 years ago, initially observed in tuberculous lungs and later linked to various inflammatory conditions [2]. Their formation is typically initiated by the presence of foreign bodies, infectious agents, or underlying inflammatory diseases. When initial phagocytosis fails to eliminate the triggering factor, a granulomatous response ensues [3]. Granulomas can be categorized based on their underlying cause (infectious or non-infectious), morphological characteristics (epithelioid, necrotic, or complex), or the rate of macrophage turnover [4,5]. Granulomas can arise from both infectious and non-infectious etiologies. Infectious causes include tuberculosis, leprosy, syphilis, fungal infections, and parasitic infestations, whereas non-infectious causes involve conditions such as sarcoidosis, Crohn’s disease, and foreign-body reactions [6]. Multinucleated giant cells (MGCs) are large histiocytic cells resulting from the fusion of monocytes. These cells are classified into distinct subtypes based on their morphology and functional roles [7]. Langhans giant cells (LGCs), characterized by a peripheral arrangement of nuclei, are frequently associated with immune granulomas, particularly in tuberculosis [3,8]. In contrast, foreign-body giant cells (FBGCs), which display irregularly scattered nuclei, form in response to non-degradable materials, including medical implants [9,10].

In the orofacial region, primary granulomatous conditions include foreign-body reactions, delayed hypersensitivity to topical agents, and idiopathic orofacial granulomatosis. Secondary causes, on the other hand, are often associated with infectious pathogens, systemic diseases such as sarcoidosis, and inflammatory bowel conditions like Crohn’s disease [11]. Tuberculosis remains a significant global infectious disease despite advancements in healthcare. Tuberculous lymphadenitis is the most common form of extrapulmonary tuberculosis, with a predilection for cervical lymph nodes [12]. The head and neck region may be affected as a secondary site following primary pulmonary tuberculosis or as a primary infection without pulmonary involvement [13,14]. Oral tuberculosis is typically associated with extrapulmonary or disseminated forms, accounting for 0.05–5% of all cases [15]. Histopathological examination commonly reveals caseating necrosis accompanied by Langhans giant cells, while Ziehl–Neelsen staining confirms the presence of acid-fast bacilli [16]. Histoplasmosis, a fungal infection caused by *Histoplasma capsulatum*, predominantly affects immunocompromised individuals and can present as painful oral ulcerations, often mimicking malignancy [17]. Grocott methenamine silver (GMS) staining highlights fungal elements, and PCR analysis aids in rapid identification [18,19]. Syphilis is a chronic sexually transmitted infection caused by *Treponema pallidum (TP)* [20]. In its tertiary stage, syphilitic granulomas, known as gummas, can develop, leading to significant tissue destruction, including palatal perforation [21,22]. Foreign materials, such as dental materials and cosmetic fillers, represent a common cause of oral granulomatous disorders [11,23]. These materials exhibit distinct histologic features, enabling their identification with a high degree of confidence [11]. Sarcoidosis is a systemic granulomatous disease that can affect multiple organs, with cervical lymphadenopathy being the most frequent manifestation in the head and neck region. Histopathological examination of lymph node biopsies typically reveals non-caseating epithelioid cell granulomas [24]. Crohn’s disease (CD) is a chronic immune-mediated disorder characterized by an inflammatory process affecting various segments of the gastrointestinal tract, most commonly the terminal ileum and proximal colon [25]. Notably, oral manifestations may precede gastrointestinal symptoms in up to 60% of affected individuals, making early recognition crucial for diagnosis [26].

Following careful evaluation of hematoxylin and eosin (H&E) sections, special stains can be utilized to enhance diagnostic sensitivity. Given that mycobacteria and fungal organisms are the most common etiological agents in granulomatous inflammation, staining techniques targeting these pathogens are prioritized. GMS and Ziehl–Neelsen (acid-fast bacilli, AFB) stains are the most frequently employed methods for detecting fungal elements and acid-fast bacilli, respectively. Additionally, periodic acid–Schiff (PAS) stain is sometimes preferred for fungal identification due to its ability to reduce background staining artifacts [27]. A thorough assessment of granulomas at multiple tissue depths is essential, as microorganisms may be missed when examining single sections or relying on low magnification. While special stains and H&E examination provide valuable diagnostic insights, mycobacterial species cannot be reliably differentiated based on morphology alone. Therefore, culture and molecular techniques are required for definitive identification [27]. Advances in molecular diagnostics, whether applied to cultured organisms or directly to fresh or formalin-fixed specimens, have significantly improved the specificity and accuracy of pathogen identification, particularly for mycobacterial species [28].Therefore, the aim of this retrospective study was to characterize the clinicopathological features of granulomatous lesions in the head and neck region, and to evaluate the diagnostic utility of histochemical stains (ZN, PAS, GMS) and real-time PCR in detecting *Mycobacterium tuberculosis* DNA in formalin-fixed, paraffin-embedded (FFPE) tissues. This integrated approach seeks to highlight the diagnostic contributions of conventional and molecular techniques in differentiating infectious and non-infectious causes of granulomatous inflammation.

## 2. Materials and Methods

### 2.1. Study Design

This retrospective cross-sectional study included 42 cases of granulomatous lesions of the head and neck, selected from 14,458 archived cases diagnosed between 1975 and 2025 at the oral Pathology Laboratory, College of Dentistry, University of Baghdad, Baghdad, Iraq [29]. All samples were (FFPE) tissues showing histopathological features of granulomatous inflammation. We obtained the necessary ethical approval from the ethics committee of the College of Dentistry at the University of Baghdad. The reference number for this ethical approval is 875, dated 3 December 2023. Of the 42 granulomatous cases studied, 25 were confirmed as tuberculosis or tuberculous lymphadenitis through comprehensive clinical follow-up at the Baghdad Tuberculosis Institute, Baghdad, Iraq. This constituted a definitive clinical diagnosis based on presentation, response to anti-tuberculous therapy, and/or microbiological data available at the time of original diagnosis. The remaining 17 were diagnosed based on clinical and pathological criteria. Chronic granulomatous inflammation and orofacial granulomatosis were diagnosed by exclusion, while foreign-body granulomas showed a characteristic histology with exogenous material. Crohn’s-related granulomas were confirmed by gastrointestinal symptoms and biopsy. Wegener’s granulomatosis was identified by necrotizing granulomas with vasculitis and referred from another center to confirm the diagnosis, and fungal granulomas were diagnosed by detecting fungal elements in giant cells. All cases were examined using H&E-stained sections by expert pathologists [30], special stains (ZN, PAS, GMS), and real-time PCR for Mycobacterium tuberculosis. Demographic and clinical data were also analyzed to support diagnosis.

### 2.2. Diagnostic Workflow and Blinding

The diagnostic workflow for this retrospective study followed standard pathology practice. The H&E diagnosis was established first and served as the baseline for selecting cases and guiding the application of ancillary tests. Consequently, the pathologists interpreting the histochemical stains were not blinded to the initial H&E findings. To ensure objectivity in the molecular analysis, DNA extraction and real-time PCR were performed on samples identified by case number, with results generated by the instrument’s software based on standardized cycle threshold cutoffs.

### 2.3. Inclusion Criteria

Cases were included if they showed histopathological features consistent with granulomatous inflammation—such as Langhans or foreign-body giant cells and epithelioid histiocytes—and were located in the head and neck region (e.g., jaw bones, oral soft tissues, or cervical lymph nodes). Only (FFPE) tissue blocks suitable for staining and molecular analysis were selected. Eligible cases were diagnosed between 1975 and 2025 and archived in the records of university of Baghdad/College of Dentistry. Sufficient clinical and demographic data, including age, sex, lesion site, and preliminary diagnosis, were also required.

### 2.4. Exclusion Criteria

Cases were excluded if the tissue was poorly preserved, lacked adequate volume for additional processing, or displayed non-specific inflammation without granulomatous features. Lesions with unknown or extra-head-and-neck anatomical sites, or cases with incomplete clinical or demographic information, were also excluded.

### 2.5. Staining Procedure

For histochemical evaluation, tissue sections were stained using ZN, PAS, and GMS methods using Stain Kit (PathnSitu, Hyderabad, India). In the ZN procedure, slides were stained with Carbol Fuchsin at 60 °C for 20 min to facilitate dye penetration into acid-fast bacilli (AFB), followed by rinsing in tap water. Decolorization was performed until the pink dye ceased to run, and slides were then washed and counterstained briefly with Light Green to provide background contrast. Dehydration through graded alcohols and clearing in xylene were followed by mounting for microscopic evaluation. For PAS staining, after baking and deparaffinization, sections were oxidized with periodic acid for 5 min to generate aldehydes, then treated with Schiff’s reagent in a dark chamber for 5–15 min. Following water rinses, slides were counterstained with modified Mayer’s hematoxylin, dehydrated in ascending alcohol concentrations, cleared in xylene, and mounted with DPX. The GMS procedure began with deparaffinization and oxidation in chromic acid at 60 °C for 10–15 min, followed by treatment with sodium metabisulfite. Sections were incubated in GMS working solution at 60 °C for 30 min, rinsed, toned with gold chloride, and treated with sodium thiosulphate to remove residual silver. Slides were then counterstained with Light Green, dehydrated, cleared in xylene, and mounted for examination.

### 2.6. Histological Criteria for Fungal Identification

Following positive staining with PAS and/or GMS, fungal organisms were morphologically categorized based on established histological criteria [31,32]. Acknowledging the potential for morphological overlap, the following features were carefully assessed for presumptive identification:

- *Aspergillus* species: Characterized by the presence of uniform, slender (3–6 µm), septate hyphae with dichotomous, acute-angle (approximately 45°) branching.
- *Paecilomyces* species: As a key mimic of *Aspergillus*, a careful search for distinguishing features was performed, including more tapered hyphal bases, irregular hyphal contours, and branching angles that can be less consistent than the classic 45° seen in *Aspergillus*.
- *Mucorales*: Identified by broad (5–20 µm), ribbon-like, pauciseptate, or non-septate hyphae with irregular contours and wide-angle, often 90°, branching.

This histological assessment provided a presumptive identification, as a definitive species-level diagnosis, especially the differentiation of *Aspergillus* from *Paecilomyces*, often requires culture or molecular analysis, which was not feasible for this retrospective study on long-term archival tissue.

### 2.7. DNA Extraction and Real-Time PCR Analysis

DNA extraction from (FFPE) tissue sections was initiated through deparaffinization using mineral oil [33], utilizing the ReliaPrep™ FFPE gDNA Miniprep System (Promega, Madison, WI, USA; Cat. No. A2351), and briefly, 300 µL of mineral oil was added to each sample, followed by incubation at 80 °C for 1 min to melt the paraffin. The mixture was then vortexed to ensure thorough emulsification. Subsequently, 200 µL of lysis buffer was added, and samples were centrifuged at 10,000× *g* for 15 s at room temperature, yielding a clear separation into an upper oil layer and a lower aqueous phase containing the biological material.

To digest proteins and release nucleic acids, 40 µL of Proteinase K was added to the aqueous phase, mixed thoroughly, and incubated at 56 °C for 1 h. This was followed by a second incubation at 80 °C for 4 h to optimize nucleic acid yield. After cooling to room temperature, samples were briefly centrifuged to collect condensate.

The extracted DNA was subjected to real-time PCR (Sacace Biotechnologies, Como, Italy) targeting the *Mycobacterium tuberculosis* complex by detecting the IS6110 gene using hybridization probes with FAM emission. The PCR protocol began with an initial activation at 95 °C for 15 min, followed by 5 cycles of denaturation (95 °C for 15 s), annealing (65 °C for 30 s), and extension (72 °C for 15 s). This was followed by 40 additional cycles with the same parameters to ensure amplification.

To control for PCR inhibition and false negatives, an internal control (IC) emitting in the JOE channel was included in each reaction. A sample was interpreted as positive for *M. tuberculosis* if it produced a fluorescence signal exceeding the threshold in the FAM (green) channel. Conversely, samples showing no FAM signal but a positive JOE signal were considered negative.

### 2.8. Statistical Analysis

Statistical analyses were conducted using SPSS software, version 26. Descriptive statistics were used to summarize continuous variables, including the mean, standard deviation, and range. Normality of the data distribution was assessed using the Shapiro–Wilk test. As the assumptions for parametric testing were met, the independent two-tailed *t*-test was used to compare means between groups. Categorical data were described using frequencies and percentages and analyzed using the Chi-square or Fisher’s exact test, as appropriate. A *p*-value less than 0.05 was considered statistically significant.

### 2.9. Analysis of Sample Age and PCR Sensitivity

To investigate the impact of sample age on PCR performance, the 25 confirmed tuberculous cases were stratified into two groups based on the year of tissue block preparation: recent samples (archived < 10 years) and older samples (archived ≥ 10 years). The rates of PCR positivity between these two groups were compared using Fisher’s exact test.

## 3. Results

### 3.1. Clinical Findings

This study included 42 patients with granulomatous lesions of the head and neck. Females comprised 59.5% of the cases (25 patients), while males accounted for 40.5% (17 patients), resulting in a male-to-female ratio of approximately 1:1.47. The age of patients ranged from 5 to 80 years, with a mean age of 36.28 ± 20.6 years. The largest age group included individuals aged 50 years and above, representing 38.1% of the cohort.

In terms of anatomical distribution, soft tissue involvement was more common, observed in 71.4% of cases, whereas bone involvement was seen in 28.6%. The mandible was the most frequently affected site (64.3%), followed by the maxilla (23.8%), with fewer cases affecting other regions such as the sinus and facial soft tissues, as in Table 1.

### 3.2. Histological Findings

Necrotizing granulomas were the predominant pattern, observed in 29 cases (69%), while non-necrotizing granulomas were identified in 13 cases (31%).

Multinucleated giant cells were a frequent feature in both types, with Langhans-type giant cells observed in 32 cases (78%). These were more commonly associated with necrotizing granulomas. Foreign-body-type giant cells were seen in nine cases (22%) and were exclusively found in non-necrotizing granulomas.

**Figure 1:**
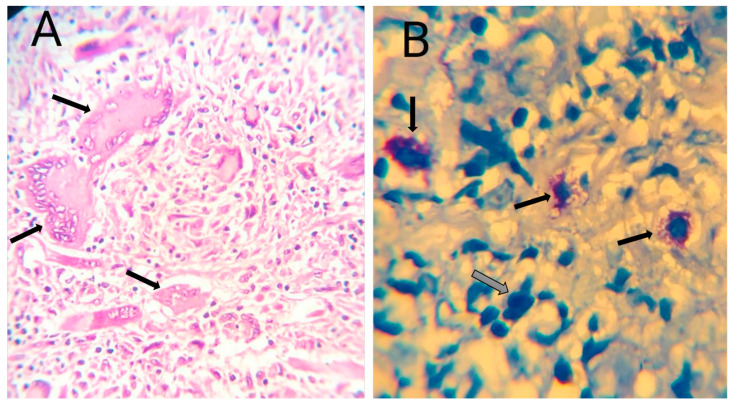
(**A**) Hematoxylin and eosin (H&E) stain of tuberculous granuloma(scale bar = 50 μm, ×400): The section shows a well-organized granulomatous inflammatory reaction composed predominantly of epithelioid histiocytes arranged in cohesive aggregates. Scattered, multinucleated, Langhans-type giant cells with peripherally arranged nuclei (black arrows) are evident within the granulomatous focus. Numerous lymphocytes and plasma cells form a peripheral inflammatory cuff surrounding the granuloma. (**B**) By using ZN stain, this micrograph demonstrates the presence of numerous acid-fast bacilli(scale bar = 10 μm, ×1000) appearing as slender, bright red, rod-shaped organisms (black arrows) distributed singly and in small clusters within the necrotic and cellular areas of the granuloma. The background tissue exhibits granular blue staining due to the counterstain, highlighting the dense inflammatory infiltrate (white arrow) and disrupted cellular architecture.

**Figure 2:**
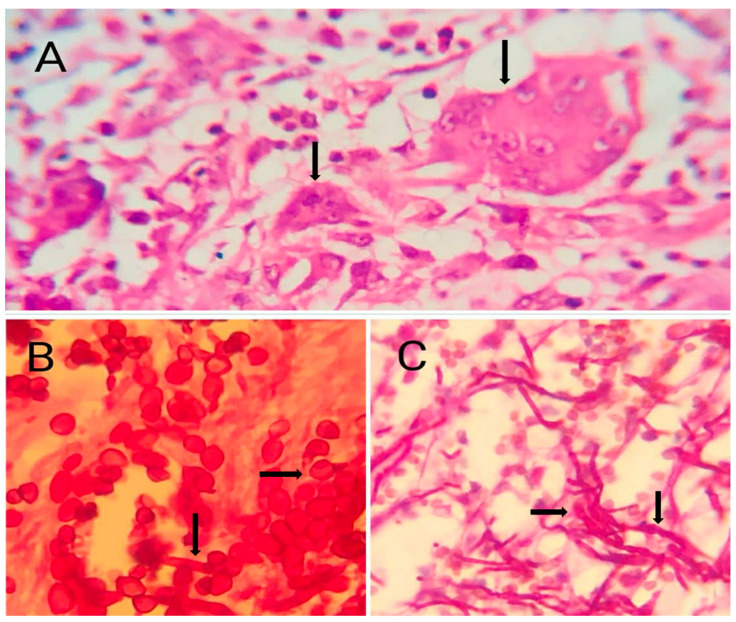
PAS stain showing fungal elements. (**A**) The section shows granulomatous inflammation with a prominent foreign-body-type giant cell containing numerous randomly distributed nuclei (black arrows). Surrounding the giant cell are epithelioid histiocytes and a mild lymphocytic infiltrate within fibrous stroma. PAS staining highlights pale magenta extracellular material. Findings are consistent with a foreign-body granulomatous reaction(scale bar = 50 μm, ×400). (**B**) This image reveals clusters of ovoid to elongated fungal organisms (black arrows) with bright magenta staining, some displaying budding suggestive of yeast forms (scale bar = 10 μm, ×1000). The background shows lightly stained connective tissue and scattered inflammatory cells. (**C**) Numerous branching, septate fungal hyphae (black arrows) stained intensely magenta against a lightly stained background. The fungal filaments are long, slender, and uniform in width, with evident septations suggestive of *Aspergillus* (scale bar = 50 μm, ×400).

**Figure 3:**
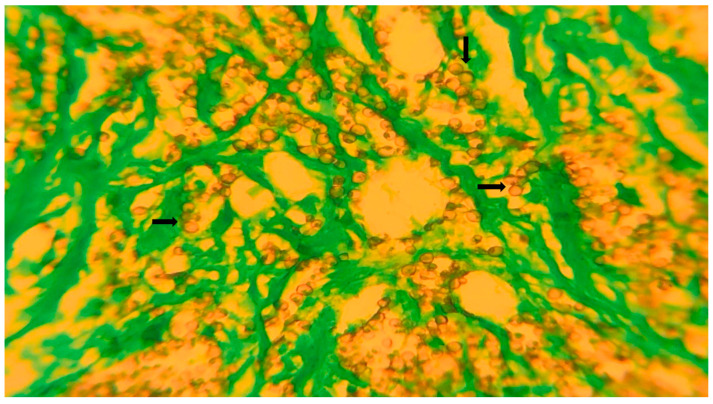
GMS stain showing fungal elements (scale bar = 50 μm, ×400). This photomicrograph demonstrates abundant round to ovoid (black arrows) fungal spores and hyphae stained black or dark brown against a bright green background, characteristic of a positive GMS reaction. The fungal walls are clearly outlined, allowing for the precise identification of morphology.

As can be seen in Table 2, special staining techniques revealed further diagnostic insights. Acid-fast bacilli were detected in seven cases using ZN (Figure 1). PAS stain was positive in five cases (Figure 2), and GMS stain was positive in four cases (Figure 3), indicating the presence of fungal organisms. Interestingly, most cases with positive fungal staining were also within the necrotizing granuloma group.

### 3.3. PCR Findings

DNA amplification using real-time PCR targeting the *Mycobacterium tuberculosis* complex (MTBC) was performed on all 42 FFPE granulomatous tissue samples. PCR was positive in 6 out of 25 tuberculous granuloma cases (24%) and negative in all non-tuberculous granulomas (0/17). Although PCR positivity was confined exclusively to the tuberculous group, the difference did not reach statistical significance (*p* = 0.066).

When granulomas were analyzed by type, PCR positivity was seen only in caseating granulomas (6/29; 20.7%) and was absent in all non-caseating granulomas (0/13). However, this association was not statistically significant (*p* = 0.153).

### 3.4. Impact of Sample Age on PCR Sensitivity

A stratified analysis of the 25 confirmed tuberculous granuloma cases revealed a strong trend linking sample archival age and PCR sensitivity. In the 15 samples archived for less than 10 years, PCR detected MTB DNA in five cases, yielding a sensitivity of 33.3% within this recent subgroup. In contrast, among the 10 samples archived for 10 years or more, only one case was positive, resulting in a sensitivity of 10.0%. Although this trend did not reach statistical significance (Fisher’s exact test, *p* = 0.35), it strongly suggests that PCR performance is degraded in older archival tissues.

## 4. Discussion

### 4.1. Demographic and Anatomical Distribution

#### 4.1.1. Age and Gender Distribution

The demographic analysis of the 42 cases revealed a female predominance, particularly in tuberculous lymphadenitis, with a male-to-female ratio of 1:1.78, consistent with findings by Museedi et al., who reported a similar ratio of 1:1.73 in head and neck tuberculous lymphadenitis [14]. Moreover, other studies have reported it to be more common in females [34,35,36,37]. In contrast, the study conducted by Bruzgielewicz et al. reported a male predominance in head and neck tuberculosis with a male-to-female ratio of 1.43:1 [38]. The observed female predominance in tuberculous granulomas is often attributed to hormonal and immunological factors, such as estrogen’s role in enhancing macrophage activity and promoting Th1 responses, which are critical in granuloma formation. Despite the abundance of research showing that estrogens reduce inflammatory responses, some studies have found that, depending on the particular cytokine or cell type examined, estrogens can have a proinflammatory or even dual pro- and anti-inflammatory effect [39]. It has been shown that E2 therapy increases the production of type-II interferon, IFN-γ [40]. Amina Belboul et al. demonstrate that estrogen can promote the alternative activation of macrophages via estrogen receptor alpha (ER-α) signaling [41]. These sex hormones may also regulate proinflammatory cytokine expression and modulate cellular responses to *Mycobacterium tuberculosis*, potentially resulting in more pronounced granulomatous reactions in females. In contrast, the distribution of non-tuberculous granulomas was nearly equal between the sexes (eight males, nine females), suggesting that these lesions—such as fungal, foreign body, or idiopathic granulomas—are more influenced by local or systemic factors unrelated to sex, supporting the absence of a significant gender predisposition in non-tuberculous etiologies.

The age distribution in this study demonstrated considerable variability, with a mean age of 36.3 years and a standard deviation of 20.6, indicating that granulomatous lesions can occur across a wide age range—from childhood to late adulthood. This finding is consistent with previous studies, such as that of Museedi et al., who reported a similar mean age of 39.5 years (SD ± 21.4) in patients with head and neck tuberculosis [14]. Similarly, Bruzgielewicz et al. found that the disease most commonly affects individuals in the 30–40-year age range [38]. These parallels highlight that granulomatous inflammation in the head and neck is not age-specific but may arise across the lifespan due to a variety of underlying etiologies.

#### 4.1.2. Anatomical Distribution

This study revealed a marked predominance of soft tissue involvement over bone in granulomatous lesions of the head and neck. Soft tissue sites—such as cervical lymph nodes, gingiva, buccal mucosa, lips, and oral submucosa—accounted for most cases, with tuberculosis being the leading cause, especially in lymphoid-rich regions. This aligns with the known predilection of extrapulmonary TB for cervical lymph nodes. The frequent soft tissue involvement in TB-related granulomas highlights the importance of maintaining a clinical suspicion of TB in persistent swelling, particularly in endemic areas or among immunocompromised patients.

In contrast, bone-involved lesions were fewer and more diverse in etiology. While some were tuberculous, others stemmed from chronic osteomyelitis, fungal infections, or systemic conditions like granulomatosis with polyangiitis. The lower frequency of skeletal TB may relate to reduced vascularity and the hematogenous nature of spread. Additionally, bone granulomas often reflect a chronic or systemic pathology.

The contrast between soft tissue and bone involvement underscores distinct pathophysiological mechanisms and diagnostic approaches—soft tissue lesions tend to present earlier and are more accessible, while osseous lesions may require imaging and histology for diagnosis.

### 4.2. Histopathological Analysis

#### 4.2.1. Granuloma Typing: Caseating and Non-Caseating Lesions

Histopathological evaluation in this study revealed a predominance of caseating granulomas, observed in 29 cases (69.0%), highlighting the central role of infectious etiologies—particularly *Mycobacterium tuberculosis*—in granulomatous inflammation of the head and neck. This lines up with several studies—particularly in pulmonary pathology—to have shown that mycobacteria and fungi are the most common infectious etiologies associated with necrotizing granulomas [42]. Caseating granulomas feature a central zone of necrosis, grossly resembling cheese, surrounded by epithelioid histiocytes, Langhans-type multinucleated giant cells, lymphocytes, and fibroblasts. This structured granulomatous architecture is a hallmark of tuberculous infection, reflecting the host’s immune attempt to contain the pathogen.

The necrotic center is thought to result from hypoxia, cytotoxic T-cell activity, and cytokine release (e.g., TNF-α, IFN-γ), which drive macrophage differentiation and granuloma maturation. Langhans giant cells, indicative of TB, are key diagnostic markers in tissue sections, especially in endemic areas or clinically suspected cases.

Non-caseating granulomas were less frequent and lacked central necrosis. Composed mainly of epithelioid histiocytes and lymphocytes—sometimes with multinucleated giant cells—these lesions are typically associated with non-infectious inflammatory conditions. Examples include sarcoidosis, Crohn’s disease, and granulomatosis with polyangiitis, where granulomas arise from immune dysregulation. Foreign-body granulomas, triggered by materials like sutures or keratin, also present with a non-caseating morphology and foreign-body-type giant cells.

#### 4.2.2. Ziehl–Neelsen Staining in Different Disorders

In the current study, (ZN) staining demonstrated limited sensitivity for detecting *Mycobacterium tuberculosis* in (FFPE) tissues from the head and neck, with acid-fast bacilli identified in only a subset of histologically confirmed tuberculosis cases. This finding is consistent with previous studies reporting low sensitivity ranges, typically from 0% to 40% [43,44]. Other studies that agree with this finding are those of Allan N. Njau et al. and Bipin Kumar, who reported that the sensitivity levels of (ZN) staining were only 20.3% and 27–60%, respectively [45,46], reflecting the diagnostic limitations of ZN in FFPE samples. These limitations are often due to factors such as a low bacillary burden, extensive necrosis, and bacillary degradation during tissue processing. In contrast, higher ZN sensitivity—up to 62%—has been reported in studies using fine-needle aspiration (FNA) samples [47], which are less affected by fixation and generally contain fresher material with a higher likelihood of bacillus detection.

The limited sensitivity of (ZN) staining in this study is likely due to the low and uneven distribution of bacilli in mycobacterial infections [48]. Fixatives and organic solvents used in tissue processing, especially xylene, can degrade mycolic acids—key targets for acid-fast dyes—reducing stain uptake and leading to false negatives [49,50]. The Fite stain, which preserves mycolic acids by minimizing xylene exposure, has been shown to be more sensitive in detecting mycobacteria [51].

Additionally, the focal presence of bacilli within granulomas means that AFB may be missed if the examined FFPE tissue section does not include the infected area [52].

Notably, 71.4% of ZN-positive cases in this study exhibited caseation necrosis, supporting findings by Majeed and Bukhari, who reported that AFB positivity is high in necrotic granulomas [53], echoed by other studies [54,55].

In contrast, a non-tuberculous cause showed no AFB positivity, suggesting alternative etiologies such as fungal infections or foreign-body reactions. This is in line with Guarner’s report that 12–36% of granulomas lack a defined cause even after histopathologic assessment [56].

#### 4.2.3. Grocott Methenamine Silver (GMS) and Periodic Acid–Schiff (PAS)

PAS and GMS are key tools in detecting fungal organisms within FFPE tissues, especially when fungal infection is suspected [57]. GMS is widely regarded as the gold standard due to its high sensitivity [58,59], while PAS is commonly used for its practicality, particularly in cases with inconclusive culture results [60,61].

In the current study, 7 out of 42 granulomatous lesions (16.7%) were identified as fungal infections, all of which were non-tuberculous. PAS demonstrated higher sensitivity than GMS under the study conditions, detecting fungal elements in most positive cases. The reduced performance of GMS may be attributed to factors like fungal viability, tissue infiltration, and morphological degradation due to long-term fixation. The findings underscore the benefit of using both PAS and GMS together to improve detection accuracy. None of the tuberculous granulomas showed PAS or GMS positivity, which is consistent with the known structural characteristics of *Mycobacterium tuberculosis*, lacking the polysaccharide-rich walls necessary for these stains. Similarly, non-fungal, non-tuberculous granulomas—such as those due to sarcoidosis or foreign-body reactions—were also negative, reaffirming the selective utility of PAS and GMS for fungal detection and the need to interpret their results in conjunction with clinical and histopathologic data. While PAS and GMS stains were invaluable for identifying the presence of fungal organisms in non-tuberculous granulomas, it is crucial to acknowledge the limitations of relying solely on histomorphology for fungal speciation. As noted in the literature, significant morphological overlap exists between different fungal genera. This is particularly relevant for the distinction between *Aspergillus* and its well-known mimic, Paecilomyces, as both present in tissue as septate hyphae with acute-angle branching, making them very difficult to distinguish reliably [32].

Although we carefully assessed for subtle distinguishing features, this distinction is not merely academic; *Paecilomyces* species can exhibit different, often higher, resistance to certain antifungal agents compared to *Aspergillus*. Therefore, while our diagnoses of “aspergillosis” are based on classic morphological patterns, the possibility of a mimic like *Paecilomyces* cannot be entirely excluded without ancillary confirmation via culture or molecular sequencing. The primary value of histochemical staining in this retrospective context remains the crucial differentiation of fungal from mycobacterial or other causes of granulomatous inflammation.

### 4.3. PCR Analysis

Real-time PCR has proven to be a highly sensitive and specific method for detecting *Mycobacterium tuberculosis (MTB)*, particularly valuable when traditional histology and staining are inconclusive [62,63]. However, its effectiveness is significantly reduced in (FFPE) tissues due to DNA fragmentation, cross-linking, and PCR inhibitors. Sensitivity in FFPE samples generally ranges between 50% and 60% [64]. In this study, the overall PCR sensitivity for tuberculosis was low at 24.0%, a finding that requires careful interpretation in the context of our unique archival cohort. Our stratified analysis strongly suggests that the archival age of the FFPE samples was a critical factor influencing molecular detection. PCR sensitivity was substantially higher in recent samples (<10 years old) at 33.3% compared to only 10.0% in older samples. This demonstrates that while PCR maintains high specificity, its diagnostic utility is significantly degraded over time, likely due to the progressive DNA fragmentation and cross-linking known to occur in long-term stored blocks, consistent with findings by Luo et al. [64]. The detection of MTB DNA in one sample from 1988 is a noteworthy outlier. It suggests that while DNA degradation is the general rule, occasional successful amplification in very old samples may be possible, perhaps in cases with an unusually high initial bacillary load or superior tissue preservation.

Other challenges include the formalin-induced cross-linking of DNA and proteins, low bacillary load in extrapulmonary lesions, and interference from PCR inhibitors like heme or immunoglobulins, particularly in blood-rich tissues [52,65,66,67,68,69]. Larger tissue samples may also introduce more inhibitors despite containing more total DNA. Additionally, prolonged fixation times intensify DNA damage, further reducing PCR efficiency [70,71]. Efforts to overcome these limitations include improved deparaffinization methods, optimized DNA extraction kits for FFPE tissues, and minimizing the fixation time using 10% neutral buffered formalin, which is preferred for preserving DNA integrity [72,73,74,75]. Nonetheless, even with these advances, FFPE tissues typically yield lower-quality DNA than fresh-frozen samples, which remain the gold standard for molecular testing. PCR success also hinges on optimizing all aspects of the protocol, including enzyme choice and reaction conditions [76,77]. Furthermore, the PCR assay employed in this investigation targets the IS6110 insertion sequence, an insertion element that is unique to members of the *Mycobacterium tuberculosis* complex (MTBC) and has thus emerged as a crucial diagnostic tool for MTBC species identification [78]. Although PCR testing showed a low sensitivity of 24% in this study, its specificity and positive predictive value reached 100%, making it a useful tool for confirming tuberculosis infection in cases with compatible histological features but negative ZN staining. PCR contributed to confirming the diagnosis in six cases, highlighting its importance in supporting the diagnosis in archival paraffin-embedded tissue samples, particularly in paucibacillary infections or those with degraded DNA due to prolonged fixation, as noted in previous studies. Therefore, PCR serves as a valuable adjunct diagnostic tool in settings where conventional tests have limited efficacy, despite its sensitivity-related limitations.

This study has several limitations inherent to its retrospective design. First, the interpretation of histochemical stains was not performed in a blinded fashion, as pathologists were aware of the baseline H&E diagnosis, which could potentially introduce observer bias. However, this workflow reflects standard clinical practice where ancillary stains are used to confirm or refute a differential diagnosis derived from the initial examination. Second, and most critically, the reliance on archival FFPE tissue, especially very old blocks, significantly impacted the sensitivity of molecular testing, and these results may not be generalizable to diagnoses made using fresh tissue samples.

## 5. Conclusions

Tuberculosis was the most common cause of granulomatous lesions in the head and neck, followed by fungal and non-specific etiologies. While histochemical stains and PCR enhanced diagnostic accuracy, ZN stain and PCR sensitivity were limited in older archival samples. Langhans-type giant cells were more frequent in tuberculous, caseating granulomas but were not exclusive to TB, serving only as supportive—not definitive—diagnostic features. These findings emphasize the importance of integrating clinical, histopathological, and molecular data for accurate diagnosis and optimal patient management.

## Figures and Tables

**Table 1:** Clinical and histopathological characteristics of granulomatous lesions including lesion category, giant cell type, granuloma pattern, and site distribution.

Variable	No. (*n* = 42)	Percentage (%)
Lesion category		
Soft tissue	30	71.4
Bone	12	28.6
Type of giant cell		
Langhans	32	78.0
Foreign body	9	22.0
Type of granuloma		
Caseating	29	69.0
Non-caseating	13	31.0
Site of lesion		
Mandibular	27	64.3
Maxillary	10	23.8
Undefined	5	11.9

Only 41 specimens were analyzed for this feature; one case diagnosed as Wegener’s granulomatosis (granulomatosis with polyangiitis) was excluded from the statistical analysis of giant-cell-type distribution due to the absence of identifiable multinucleated giant cells in the examined histological sections. The granulomatous inflammation in this case was poorly formed and lacked sufficient morphological features for definitive classification of the giant cell subtype.

**Table 2:** Combined granuloma classification and diagnostic technique positivity by etiology.

Diagnosis	Necrotizing (*n*)	Non-Necrotizing (*n*)	Total (*n*)	Soft Tissue	Bone	Mandible	Maxilla	Other Site	ZN+ (*n*)	PAS+ (*n*)	GMS+ (*n*)	PCR+ (*n*)
TB	21	4	25	22	3	19	2	4	7	0	0	6
Mixed Fungal Infection	6	1	7	2	5	2	5	0	0	5	4	0
Wegener Disease	1	0	1	0	1	0	1	0	0	0	0	0
Chronic Granulomatous Inflammation	1	2	3	3	0	2	1	0	0	0	0	0
Foreign-Body Granuloma	0	3	3	1	2	2	1	0	0	0	0	0
Orofacial Granuloma	0	1	1	1	0	1	0	0	0	0	0	0
Suggestion of Crohn’s Disease	0	1	1	1	0	0	0	1	0	0	0	0
Chronic Osteomyelitis	0	1	1	0	1	1	0	0	0	0	0	0
Total	29	13	42	30	12	27	10	5	7	5	4	6

## Data Availability

The data presented in this study are available on request from the corresponding author due to privacy or ethical restrictions related to participant data.

## References

[1] Adams D.O. (1976). The granulomatous inflammatory response. A review. Am. J. Pathol..

[2] Ramakrishnan L. (2012). Revisiting the role of the granuloma in tuberculosis. Nat. Rev. Immunol..

[3] Pagán A.J., Ramakrishnan L. (2018). The Formation and Function of Granulomas. Annu. Rev. Immunol..

[4] Spector W.G. (1969). The granulomatous inflammatory exudate. Int. Rev. Exp. Pathol..

[5] Ohshimo S., Guzman J., Costabel U., Bonella F. (2017). Differential diagnosis of granulomatous lung disease: Clues and pitfalls: Number 4 in the Series “Pathology for the clinician” Edited by Peter Dorfmüller and Alberto Cavazza. Eur. Respir. Rev..

[6] Boros D.L.J.G.I., Cellular I., Mechanisms M. (2003). The cellular immunological aspects of the granulomatous response. Granulomatous Infections and Inflammations: Cellular and Molecular Mechanisms.

[7] Shrestha A., Marla V., Shrestha S., Neupane M. (2014). Giant cells and giant cell lesions of oral cavity—A review. Cumhur. Dent. J..

[8] Anderson J.M. (2000). Multinucleated giant cells. Curr. Opin. Hematol..

[9] Wang H., Jiang H., Teles R.M.B., Chen Y., Wu A., Lu J., Chen Z., Ma F., Pellegrini M., Modlin R.L. (2020). Cellular, Molecular, and Immunological Characteristics of Langhans Multinucleated Giant Cells Programmed by IL-15. J. Investig. Dermatol..

[10] Sakai H., Okafuji I., Nishikomori R., Abe J., Izawa K., Kambe N., Yasumi T., Nakahata T., Heike T. (2012). The CD40-CD40L axis and IFN-γ play critical roles in Langhans giant cell formation. Int. Immunol..

[11] Müller S. (2019). Non-infectious Granulomatous Lesions of the Orofacial Region. Head Neck Pathol..

[12] Radhi S.M.Z., Al-Nakib L.H., Gabash K.M. (2017). The Value of Ultrasonography in the Diagnosis and Evaluation of Early Therapeutic Response of Cervical Tuberculous Lymphadenitis. J. Baghdad Coll. Dent..

[13] Yang Z., Kong Y., Wilson F., Foxman B., Fowler A.H., Marrs C.F., Cave M.D., Bates J.H. (2004). Identification of risk factors for extrapulmonary tuberculosis. Clin. Infect. Dis..

[14] Museedi O., Hameedi A., Al-Dorbie B., Abdullah B. (2020). A Clinicopathologic Review of 21 Cases of Head and Neck Primary Tuberculosis. J. Oral Maxillofac. Surg..

[15] Mignogna M.D., Muzio L.L., Favia G., Ruoppo E., Sammartino G., Zarrelli C., Bucci E. (2000). Oral tuberculosis: A clinical evaluation of 42 cases. Oral Dis..

[16] Martin G., Lazarus A. (2000). Epidemiology and diagnosis of tuberculosis. Recognition of at-risk patients is key to prompt detection. Postgrad. Med..

[17] O’Connell Ferster A.P., Jaworek A., Hu A. (2018). Histoplasmosis of the head and neck in the immunocompetent patient: Report of 2 cases. Ear Nose Throat J..

[18] Wheat L.J., Freifeld A.G., Kleiman M.B., Baddley J.W., McKinsey D.S., Loyd J.E., Kauffman C.A., Infectious Diseases Society of America (2007). Clinical practice guidelines for the management of patients with histoplasmosis: 2007 update by the Infectious Diseases Society of America. Clin. Infect. Dis..

[19] Yang B., Lu L., Li D., Liu L., Huang L., Chen L., Tang H., Wang L. (2013). Colonic involvement in disseminated histoplasmosis of an immunocompetent adult: Case report and literature review. BMC Infect. Dis..

[20] Forrestel A.K., Kovarik C.L., Katz K.A. (2020). Sexually acquired syphilis: Historical aspects, microbiology, epidemiology, and clinical manifestations. J. Am. Acad. Dermatol..

[21] Leão J.C., Gueiros L.A., Porter S.R. (2006). Oral manifestations of syphilis. Clinics.

[22] McNamara M., Yingling C. (2020). The Reemergence of Syphilis: Clinical Pearls for Consideration. Nurs. Clin. N. Am..

[23] Alcântara C.E.P., Noronha M.S., Cunha J.F., Flores I.L., Mesquita R.A. (2018). Granulomatous reaction to hyaluronic acid filler material in oral and perioral region: A case report and review of literature. J. Cosmet. Dermatol..

[24] Chen H.C., Kang B.H., Lai C.T., Lin Y.S. (2005). Sarcoidal granuloma in cervical lymph nodes. J. Chin. Med. Assoc..

[25] Abbas Z.K., Zaidan T.F. (2018). The Study of Oral Findings, Oxidative Stress and Antioxidant Vitamin E in Serum and Saliva of Crohn’s Patients on Azathioprine Monotherapy and those on Combination of Anti-TNF-Î±Plus Azathioprine. J. Baghdad Coll. Dent..

[26] Lankarani K.B., Sivandzadeh G.R., Hassanpour S. (2013). Oral manifestation in inflammatory bowel disease: A review. World J. Gastroenterol..

[27] Shah K.K., Pritt B.S., Alexander M.P. (2017). Histopathologic review of granulomatous inflammation. J. Clin. Tuberc. Other Mycobact. Dis..

[28] Caulfield A.J., Wengenack N.L. (2016). Diagnosis of active tuberculosis disease: From microscopy to molecular techniques. J. Clin. Tuberc. Other Mycobact. Dis..

[29] Al-Qazzaz H.H., Abdullah B.H., Jany S.J. (2024). A clinicopathological analysis of 151 odontogenic tumors based on new WHO classification 2022: A retrospective cross-sectional study. J. Baghdad Coll. Dent..

[30] Shareef K.N., Abdullah B.H. (2022). Clinicopathological analysis of 80 cases of oral lobular and non lobular capillary hemangioma (pyogenic granuloma): A Retrospective study. J. Baghdad Coll. Dent..

[31] Broadwater D.R., Messersmith L.M., Bishop B.N., Tomkovich A.M., Salinas J.R., Lynch D.T. (2022). Development and Validation of Ultra-Rapid Periodic Acid-Schiff Stain for Use in Identifying Fungus on Frozen Section. Arch. Pathol. Lab. Med..

[32] Sowmya S.V., Augustine D., Hemanth B., Prathab A.G., Alamoudi A., Bahammam H.A., Bahammam S.A., Bahammam M.A., Haragannavar V.C., Prabhu S. (2022). Alternate Special Stains for the Detection of Mycotic Organisms in Oral Cyto-Smears-A Histomorphometric Study. Microorganisms.

[33] Mohsin S.F., Al-Drobie B. (2023). Human papillomavirus expression in relation to biological behavior, Ki-67 proliferative marker, and P53 prognostic marker in Schneiderian papilloma. J. Med. Life.

[34] Fontanilla J.M., Barnes A., von Reyn C.F. (2011). Current diagnosis and management of peripheral tuberculous lymphadenitis. Clin. Infect. Dis..

[35] Chan-Yeung M., Noertjojo K., Chan S.L., Tam C.M. (2002). Sex differences in tuberculosis in Hong Kong. Int. J. Tuberc. Lung Dis..

[36] Khan R., Harris S.H., Verma A.K., Syed A. (2009). Cervical lymphadenopathy: Scrofula revisited. J. Laryngol. Otol..

[37] Tektaş N., Yüce İ., Kara İ., Kaya M.C., Çağlı S., Gülmez E., Canöz Ö (2024). Head and Neck Manifestation of Tuberculosis. J. Clin. Pract. Res..

[38] Bruzgielewicz A., Rzepakowska A., Osuch-Wójcikewicz E., Niemczyk K., Chmielewski R. (2014). Tuberculosis of the head and neck-epidemiological and clinical presentation. Arch. Med. Sci..

[39] Dragin N., Nancy P., Villegas J., Roussin R., Le Panse R., Berrih-Aknin S. (2017). Balance between Estrogens and Proinflammatory Cytokines Regulates Chemokine Production Involved in Thymic Germinal Center Formation. Sci. Rep..

[40] Nakaya M., Tachibana H., Yamada K. (2006). Effect of estrogens on the interferon-gamma producing cell population of mouse splenocytes. Biosci. Biotechnol. Biochem..

[41] Belboul A., Ashworth J., Fadel A., Mcloughlin J., Mahmoud A., El Mohtadi M. (2025). Estrogen induces the alternative activation of macrophages through binding to estrogen receptor-alpha. Exp. Mol. Pathol..

[42] Nazarullah A., Nilson R., Maselli D.J., Jagirdar J. (2015). Incidence and aetiologies of pulmonary granulomatous inflammation: A decade of experience. Respirology.

[43] Goel M.M., Budhwar P. (2007). Immunohistochemical localization of mycobacterium tuberculosis complex antigen with antibody to 38 kDa antigen versus Ziehl Neelsen staining in tissue granulomas of extrapulmonary tuberculosis. Indian J. Tuberc..

[44] Karimi S., Shamaei M., Pourabdollah M., Sadr M., Karbasi M., Kiani A., Bahadori M. (2014). Histopathological findings in immunohistological staining of the granulomatous tissue reaction associated with tuberculosis. Tuberc. Res. Treat..

[45] Njau A.N., Gakinya S.M., Sayed S., Moloo Z. (2019). Xpert(®) MTB/RIF assay on formalin-fixed paraffin-embedded tissues in the diagnosis of extrapulmonary tuberculosis. Afr. J. Lab. Med..

[46] Kumar B. (2011). Tuberculosis of the oral cavity affecting alveolus: A case report. Case Rep. Dent..

[47] Wright C.A., van der Burg M., Geiger D., Noordzij J.G., Burgess S.M., Marais B.J. (2008). Diagnosing mycobacterial lymphadenitis in children using fine needle aspiration biopsy: Cytomorphology, ZN staining and autofluorescence—Making more of less. Diagn. Cytopathol..

[48] Crothers J.W., Laga A.C., Solomon I.H. (2021). Clinical Performance of Mycobacterial Immunohistochemistry in Anatomic Pathology Specimens. Am. J. Clin. Pathol..

[49] Fukunaga H., Murakami T., Gondo T., Sugi K., Ishihara T. (2002). Sensitivity of acid-fast staining for Mycobacterium tuberculosis in formalin-fixed tissue. Am. J. Respir. Crit. Care Med..

[50] Minnikin D.E., Minnikin S.M., Parlett J.H., Goodfellow M., Magnusson M. (1984). Mycolic acid patterns of some species of Mycobacterium. Arch. Microbiol..

[51] Cocito C., Delville J. (1985). Biological, chemical, immunological and staining properties of bacteria isolated from tissues of leprosy patients. Eur. J. Epidemiol..

[52] Bao J.R., Clark R.B., Master R.N., Shier K.L., Eklund L.L. (2018). Acid-fast bacterium detection and identification from paraffin-embedded tissues using a PCR-pyrosequencing method. J. Clin. Pathol..

[53] Majeed M.M., Bukhari M.H. (2011). Evaluation for granulomatous inflammation on fine needle aspiration cytology using special stains. Pathol. Res. Int..

[54] Bezabih M., Mariam D.W., Selassie S.G. (2002). Fine needle aspiration cytology of suspected tuberculous lymphadenitis. Cytopathology.

[55] Ahmad S.S., Akhtar S., Akhtar K., Naseem S., Mansoor T. (2005). Study of fine needle aspiration cytology in lymphadenopathy with special reference to acid-fast staining in cases of tuberculosis. JK Sci..

[56] Guarner J. (2012). Detection of microorganisms in granulomas that have been formalin-fixed: Review of the literature regarding use of molecular methods. Scientifica.

[57] Shalin S.C., Ferringer T., Cassarino D.S. (2020). PAS and GMS utility in dermatopathology: Review of the current medical literature. J. Cutan. Pathol..

[58] Gomori G. (1952). Microscopic Histochemistry: Principles and Practice.

[59] Grocott R.G. (1955). A stain for fungi in tissue-sections and smears using Gomori’s methenamine-silver nitrate technic. Am. J. Clin. Pathol..

[60] Minckler D., Small K.W., Walsh T.J. (2014). Clinical and pathologic features of Bipolaris endophthalmitis after intravitreal triamcinolone. JAMA Ophthalmol..

[61] Brown R.W. (2009). Histologic Preparations: Common Problems and Their Solutions.

[62] Takahashi T., Nakayama T. (2006). Novel technique of quantitative nested real-time PCR assay for Mycobacterium tuberculosis DNA. J. Clin. Microbiol..

[63] Hillemann D., Galle J., Vollmer E., Richter E. (2006). Real-time PCR assay for improved detection of Mycobacterium tuberculosis complex in paraffin-embedded tissues. Int. J. Tuberc. Lung Dis..

[64] Luo R.F., Scahill M.D., Banaei N. (2010). Comparison of single-copy and multicopy real-time PCR targets for detection of Mycobacterium tuberculosis in paraffin-embedded tissue. J. Clin. Microbiol..

[65] Williams C., Pontén F., Moberg C., Söderkvist P., Uhlén M., Pontén J., Sitbon G., Lundeberg J. (1999). A high frequency of sequence alterations is due to formalin fixation of archival specimens. Am. J. Pathol..

[66] Surat G., Wallace W.A., Laurenson I.F., Seagar A.L. (2014). Rapid real-time PCR for detection of Mycobacterium tuberculosis complex DNA in formalin-fixed paraffin embedded tissues: 16% of histological ‘sarcoid’ may contain such DNA. J. Clin. Pathol..

[67] Chawla K., Gupta S., Mukhopadhyay C., Rao P.S., Bhat S.S. (2009). PCR for M. tuberculosis in tissue samples. J. Infect. Dev. Ctries..

[68] Akane A., Matsubara K., Nakamura H., Takahashi S., Kimura K. (1994). Identification of the heme compound copurified with deoxyribonucleic acid (DNA) from bloodstains, a major inhibitor of polymerase chain reaction (PCR) amplification. J. Forensic Sci..

[69] Al-Soud W.A., Jönsson L.J., Râdström P. (2000). Identification and characterization of immunoglobulin G in blood as a major inhibitor of diagnostic PCR. J. Clin. Microbiol..

[70] Ben-Ezra J., Johnson D.A., Rossi J., Cook N., Wu A. (1991). Effect of fixation on the amplification of nucleic acids from paraffin-embedded material by the polymerase chain reaction. J. Histochem. Cytochem..

[71] Patzina R.A., de Andrade H.F., Jr de Brito T., Filho H.C., Kauffman M.R., Pagliari C., Lucena A., da Matta V.L.R., Duarte M.I.S. (2002). Molecular and standard approaches to the diagnosis of mycobacterial granulomatous lymphadenitis in paraffin-embedded tissue. Lab. Investig..

[72] Martinez R.M. (2014). Genes in your tissue: Probe identification and sequencing microbial targets from Formalin-Fixed, Paraffin-Embedded tissue. Clin. Microbiol. Newsl..

[73] Srinivasan M., Sedmak D., Jewell S. (2002). Effect of fixatives and tissue processing on the content and integrity of nucleic acids. Am. J. Pathol..

[74] Benchekroun M., DeGraw J., Gao J., Sun L., von Boguslawsky K., Leminen A., Andersson L.C., Heiskala M. (2004). Impact of fixative on recovery of mRNA from paraffin-embedded tissue. Diagn. Mol. Pathol..

[75] Talaulikar D., Gray J.X., Shadbolt B., McNiven M., Dahlstrom J.E. (2008). A comparative study of the quality of DNA obtained from fresh frozen and formalin-fixed decalcified paraffin-embedded bone marrow trephine biopsy specimens using two different methods. J. Clin. Pathol..

[76] Greer C.E., Peterson S.L., Kiviat N.B., Manos M.M. (1991). PCR amplification from paraffin-embedded tissues. Effects of fixative and fixation time. Am. J. Clin. Pathol..

[77] Shibata D. (1994). Extraction of DNA from paraffin-embedded tissue for analysis by polymerase chain reaction: New tricks from an old friend. Hum. Pathol..

[78] Coros A., DeConno E., Derbyshire K.M. (2008). IS6110, a Mycobacterium tuberculosis complex-specific insertion sequence, is also present in the genome of Mycobacterium smegmatis, suggestive of lateral gene transfer among mycobacterial species. J. Bacteriol..

